# Assessment of Biotechnologically Important Filamentous Fungal Biomass by Fourier Transform Raman Spectroscopy

**DOI:** 10.3390/ijms22136710

**Published:** 2021-06-23

**Authors:** Simona Dzurendová, Volha Shapaval, Valeria Tafintseva, Achim Kohler, Dana Byrtusová, Martin Szotkowski, Ivana Márová, Boris Zimmermann

**Affiliations:** 1 Faculty of Science and Technology, Norwegian University of Life Sciences, P.O. Box 5003, 1432 Ås, Norway; simona.dzurendova@nmbu.no (S.D.); volha.shapaval@nmbu.no (V.S.); valeria.tafintseva@nmbu.no (V.T.); achim.kohler@nmbu.no (A.K.); dana.byrtusova@nmbu.no (D.B.); 2 Faculty of Chemistry, Brno University of Technology, Purkyňova 464/118, 61200 Brno, Czech Republic; xcszotkowski@fch.vut.cz (M.S.); marova@fch.vut.cz (I.M.)

**Keywords:** oleaginous microorganisms, biodiesel, pigments, biopolymers, carotenoids, fatty acids, chitin, chitosan, fermentation, fungi

## Abstract

Oleaginous filamentous fungi can accumulate large amount of cellular lipids and biopolymers and pigments and potentially serve as a major source of biochemicals for food, feed, chemical, pharmaceutical, and transport industries. We assessed suitability of Fourier transform (FT) Raman spectroscopy for screening and process monitoring of filamentous fungi in biotechnology. Six Mucoromycota strains were cultivated in microbioreactors under six growth conditions (three phosphate concentrations in the presence and absence of calcium). FT-Raman and FT-infrared (FTIR) spectroscopic data was assessed in respect to reference analyses of lipids, phosphorus, and carotenoids by using principal component analysis (PCA), multiblock or consensus PCA, partial least square regression (PLSR), and analysis of spectral variation due to different design factors by an ANOVA model. All main chemical biomass constituents were detected by FT-Raman spectroscopy, including lipids, proteins, cell wall carbohydrates, and polyphosphates, and carotenoids. FT-Raman spectra clearly show the effect of growth conditions on fungal biomass. PLSR models with high coefficients of determination (0.83–0.94) and low error (approximately 8%) for quantitative determination of total lipids, phosphates, and carotenoids were established. FT-Raman spectroscopy showed great potential for chemical analysis of biomass of oleaginous filamentous fungi. The study demonstrates that FT-Raman and FTIR spectroscopies provide complementary information on main fungal biomass constituents.

## 1. Introduction

Filamentous fungi have been commercially used in biotechnology for over a century, creating a range of products from organic acids, enzymes, and oleochemicals to antibiotics, statins, and steroids for applications in the food, pharma, and chemical industry [1,2,3]. Some of the most important filamentous fungal cell factories, such as *Mortierella*, *Mucor*, *Rhizopus,* and *Umbelopsis* genera, belong to the Mucoromycota taxon. Mucoromycota have gained interest due to their versatile metabolism that enables fermentation process on a wide range of feedstock, such as waste and rest materials [4,5]. When cultivated in a carbon-rich and nitrogen-limited growth conditions, Mucoromycota fungi can accumulate high amount of lipids, up to 85% of dry weight [6]. Fermentation is the most complex individual process within biotech-manufacturing and it poses a number of challenges related to productivity and quality. The main challenges are related to the variability and heterogeneity of a fermentation growth medium and to the variability in the cellular population, such as natural population heterogeneity [7]. Thus, online monitoring of fermentation is a crucial requirement for an efficient bioprocess. Unfortunately, process monitoring is still dependent on a limited number of standard sensors for pH, temperature, and gases, while the critical process parameters, such as biomass, product and substrate concentrations, and compositions, are rarely assessable on-line. Thus, there is a need for rapid methods that provide detailed chemical information for bioprocess monitoring and optimization. Process optimization and monitoring will greatly benefit from advanced spectroscopy-based sensors that will enable real-time monitoring and control of bioprocesses.

Vibrational spectroscopy, comprising of infrared and Raman spectroscopies, is considered as a rapid, inexpensive, and highly sensitive method for analysis of biological samples [8,9]. These techniques are excellent for obtaining comprehensive and detailed information in biotechnology since they can simultaneously measure broad chemical profiles of the chemical constituents present in the bioprocess via detection of numerous functional groups [10,11,12,13,14,15,16,17]. This rich spectroscopic data is interpreted by using chemometrics, classical machine learning, and deep learning methods [18,19,20,21,22,23,24]. Although most of the studies involving filamentous fungi and yeasts have been conducted by Fourier transform infrared (FTIR) spectroscopy, as of late, Raman spectroscopy has been applied to study fungi, in particular regarding pigments [12,25,26,27,28,29,30,31,32], lipids [10,29,33,34,35,36], and cell wall composition [37,38]. Compared to FTIR spectroscopy, Raman spectroscopy is based on a fundamentally different principle. While infrared spectroscopy relies on absorption of light by molecules, Raman spectroscopy is based on an inelastic Raman scattering phenomenon. Moreover, a molecule must undergo a change in dipole moment during a vibration in order for a vibrational transition to be infrared active, while a molecule must undergo a change in polarizability during a vibration for a vibrational transition to be Raman active. For this reason, Raman spectroscopy provides more information on molecular substructures containing non-polar chemical bonds, such as S-S or C=C, while infrared spectroscopy provides more information on molecular substructures containing polar chemical bonds, such as S-O or C=O. In Raman spectroscopy, molecular vibrations originate from the interaction of the sample and the excitation radiation, typically from a laser in the ultraviolet, visible, or near-infrared region of the electromagnetic spectrum. In the case of biological samples, the resulting Raman spectrum usually displays a broad range of signals related to various types of cellular analytes, such as lipids, proteins, pigments, and carbohydrates [27,35,37,39]. Raman spectroscopy is very suitable for biotechnology applications since it does not require special sample pretreatment, it is non-destructive, and it is fast. Unlike FTIR (mid-IR) spectroscopy, Raman spectroscopy is not hindered by water and glass, which is extremely useful property for application of the technique in biotechnology. Moreover, it is very versatile, from in-situ monitoring of bioprocesses in bioreactors by a Raman fiber-optic probe [40], to the detailed cellular imaging by a confocal Raman microscope [36]. The advances in Raman instrumentation, in combination with multivariate data analysis, have shown the potential of this technique in bioprocess monitoring [25,41,42] and rapid identification and classification of fungal species [10,43,44,45,46,47].

In general, Raman scattering intensities are weak and thus it is difficult to detect molecules that are not present in high concentration in the sample. However, if the excitation radiation is in resonance with the electronic transitions, a so called resonance Raman effect will occur. In that case, the Raman scattering will be tremendously enhanced, enabling detection of molecules present in relatively low concentrations. This is often the case of certain pigments, such as carotenoids, enabling measurement of analytes that are undetectable by FTIR [48]. Unfortunately, in addition to Raman and resonant Raman effect, excitation laser can often create resonance fluorescence effect. The fluorescence effect occurs when the energy of the excitation photon is close to the transition energy between two electronic states. The presence of intensive fluorescence can significantly hamper detection of the Raman effect. Another common problem in Raman spectroscopy is sample heating that leads to emission of longer-wavelength radiation and thermal interference to the Raman spectrum, and can even result in thermal degradation of the sample. Both fluorescence and thermal interferences can be minimized by using different excitation lasers, with simultaneous optimization of the Raman effect [49,50]. In general, electronic transitions are weaker at longer wavelengths, and thus detrimental effects can be avoided by the use of near-infrared (NIR) lasers, such as neodymium doped yttrium aluminum garnet (Nd:YAG) laser with excitation at 1064 nm. Moreover, use of such a long-wavelength excitation laser can significantly increase penetration depth, compared to visible (short-wavelength) lasers, thus allowing more comprehensive analysis of a sample [51]. However, NIR excitation lasers offer significantly lower Raman sensitivity compared to ultraviolet and visible lasers, and thus they often require Fourier transform (FT) Raman spectrometers with a Michelson interferometer and a FT processor for signal enhancement. In the last decade, FT-Raman spectroscopy gained momentum in analyses of biological samples [52,53,54,55]. However, FT-Raman spectroscopy remains unexplored in the analysis of filamentous fungi, although the potential of this technique for chemical characterization of filamentous fungi was demonstrated almost three decades ago by Edwards et al. [39]. In the meantime, only one other study, with a limited focus on cinnabarin production by *Pycnoporus sanguineus*, has been conducted [30].

A number of our studies have shown that FTIR spectroscopy can be used for chemical characterization of Mucoromycota fungi [56,57,58,59]. The Duetz-microtiter plate system (Duetz-MTPS) for microbial cultivation, in combination with FTIR spectroscopy and multivariate data analysis, can be used as a powerful high-throughput low-cost method for the screening of filamentous fungi for biotechnological production of various biochemicals, such as single cell lipids, polyphosphates, and polyglucosamines (chitin and chitosan) [56]. In this study, we assessed the potential of FT-Raman spectroscopy for chemical characterization of biomass of Mucoromycota filamentous fungi in biotechnology research and production. Moreover, the same sample set was measured by FTIR spectroscopy and high performance liquid chromatography (HPLC) for pigment analysis, gas chromatography (GC) for lipid analysis, and assay-based ultraviolet–visible (UV/VIS) spectroscopy and nuclear magnetic resonance (NMR) spectroscopy for analysis of cellular phosphorus. Thus, advantages and disadvantages of FT-Raman spectroscopy over FTIR spectroscopy were evaluated in respect to various chemical constituents in the fungal biomass, such as lipids, proteins, cell wall carbohydrates, polyphosphates, and carotenoid pigments.

## 2. Results and Discussion

### 2.1. Chemical Composition of Fungal Biomass

**Figure 1:**
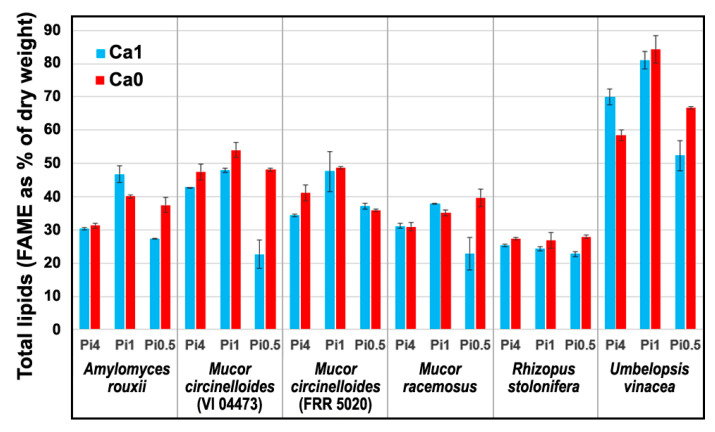
Total lipids content of the fungal samples grown under six different conditions (average values and range based on measurements of two biological replicates).

The fungal samples, belonging to the subset of samples presented in our previous study [60], were selected for the vibrational spectroscopy study due to their high variation in chemical composition. The selected oleaginous filamentous fungi were identified as a potentially good producers of valuable metabolites, such as lipids, carbohydrates (chitin, chitosan and beta-glucan), polyphosphates and carotenoid pigments [56,59]. Under nitrogen-limitation these fungi accumulate lipids in the form of free fatty acids and their derivatives, such as acylglycerols and glycerophospholipids, where triacylglycerols make by far the biggest fraction [61]. For all samples, determination of total lipids (as fatty acid methyl esters) was conducted by direct transesterification and GC-FID analysis, as reported in our previous study [60]. The samples contained the following range of amounts of the total lipids (expressed as a percentage of a dry weight): *Amylomyces rouxii* 27–49%, *Mucor circinelloides* (strain VI 04473) 20–49%, *Mucor circinelloides* (strain FRR 5020) 34–52%, *Mucor racemosus* 19–41%, *Rhizopus stolonifera* 22–28%, and *Umbelopsis vinacea* 49–83% (Figure 1).

Moreover, accumulation of other metabolites in the fungal biomass was influenced by changing concentrations of phosphate and calcium ions in the media. The samples contained the following range of amounts of the total phosphorus (expressed as a percentage of a dry weight): *Amylomyces rouxii* 2.65–6.24%, *Mucor circinelloides* (strain VI 04473) 1.40–4.91%, *Mucor circinelloides* (strain FRR 5020) 1.86–4.50%, *Mucor racemosus* 2.28–5.20%, *Rhizopus stolonifera* 2.70–4.13%, and *Umbelopsis vinacea* 0.64–1.37% [60]. NMR spectroscopy measurements have shown that the majority of phosphorus in *Mucor circinelloides* VI 04473 samples were accumulated in the form of polyphosphates [60]. These results correspond to previously reported studies that have shown average polyphosphate accumulation of Mucoromycota biomass within 0.31–0.93% range [62], with higher accumulation for *Mucor* strains, approximately within 4–7% range [63]. Our previous studies have indicated that some Mucoromycota strains have extensive polyphosphate accumulation in non-acidic growth conditions [56,60,64]. In growth media lacking calcium, there is a decrease in polyphosphate accumulation [60], which is related to the formation of acidocalcisomes granules (polyphosphate granules), which is a calcium dependent process [60,65]. In the formation of acidocalcisomes calcium and other cations functioning as a neutralizing agent for neutralizing negative charge of polyphosphate molecules, therefore calcium availability is important prerequisite for the formation of polyphosphate granules.

In our previous study, we have observed that *Amylomyces*, *Mucor*, and *Rhizopus* can overproduce chitin/chitosan under low phosphate growth conditions [56]. Cell wall of Mucoromycota fungi is typically composed of fibrillar, rigid, and shape determining polyglucosamines, in particular chitin, chitosan, and chitin–glucan complexes. These carbohydrates are embedded in an amorphous matrix of glucans and glycoproteins, and, in some cases, substructures of glucuronans and polyphosphates [66,67,68]. One of the main functions of the cell wall is protection against environmental stress [68], such as acidic stress that was present in our study under low phosphate growth conditions [56,60,69]. More specifically, limitation of phosphates availability in the growth media, in combination with ammonium sulphate as a nitrogen source, leads to acidity of the growth media, and the subsequent acidic stress results with overproduction of chitin/chitosan in the fungal cell walls. Calcium is directly involved in chitin synthesis as it activates the chitin synthase enzyme in fungi [70].

**Figure 2:**
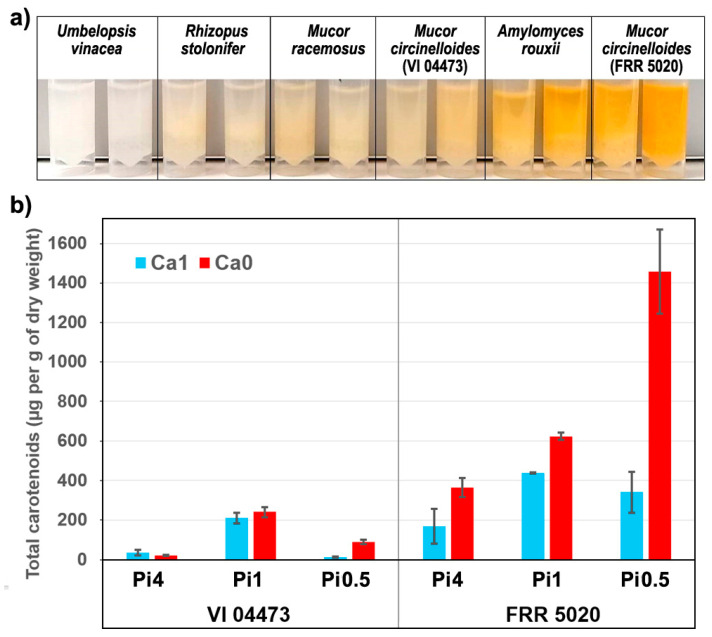
(**a**) Image of disintegrated fungal biomass of samples grown under Pi0.5 condition, with (Ca1, tube1) and without (Ca0, tube 2) calcium. (**b**) Total carotenoid content of the two *Mucor circinelloides* strains grown under six different conditions (average values and range based on measurements of two biological replicates).

In addition to changes in polyphosphates accumulation, some fungal strains have shown the influence of media nutrients on carotenoid production (Figure 2a). HPLC analysis of carotenoid content of biomasses of two *Mucor circinelloides* strains shows significant change in carotenoid production under different growth conditions. In particular, *Mucor circinelloides* strain FRR 5020, shows approximately a tenfold increase in the production of carotenoids, with accumulation of 0.14%_dry weight_ (1457 µg/g_dry weight_) total carotenoids in growth media with low phosphate and absence of calcium (Figure 2b). *Mucor circinelloides* was reported as a good candidate for carotenoid production [71], with the production of 98–378 µg/g_dry weight_ [72] (reference). The previously reported carotenoid production for other species covered by our study were 192 *Amylomyces rouxii* and 50–200 µg/g_dry weight_ for *Rhizopus stolonifer* [72]. In addition, several studies have shown that elevated temperature and light intensity will result with higher production of carotenoids in *Mucor* fungi [72,73]. Since our result for *Mucor circinelloides* strain FRR 5020 shows exceedingly high carotenoid production compared to other non-GMO Mucoromycota fungi, it strongly indicates that calcium and phosphates concentrations and acidic stress should be taken into account in carotenoid-production studies, alongside temperature and light conditions.

### 2.2. FT-Raman Chemical Profiling of Fungal Biomass

**Figure 3:**
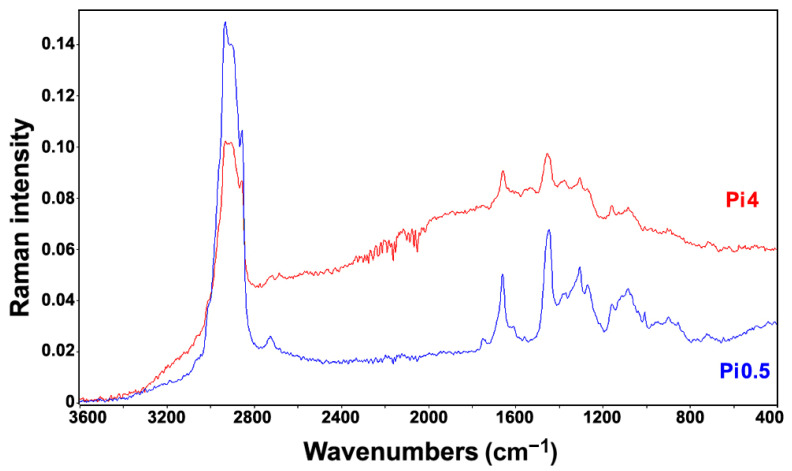
FT-Raman spectra of *Rhizopus stolonifer* cultivated under Ca1 condition and two different phosphate concentrations. The spectrum of the sample cultivated under high phosphate concentration (Pi4, red) shows a significant heating effect resulting with a distorted baseline even when measured under low excitation laser power (200 mW), compared to the spectrum of the sample cultivated under low phosphate concentration (Pi0.5, blue), which was measured under the standard laser power (500 mW).

**Figure 4:**
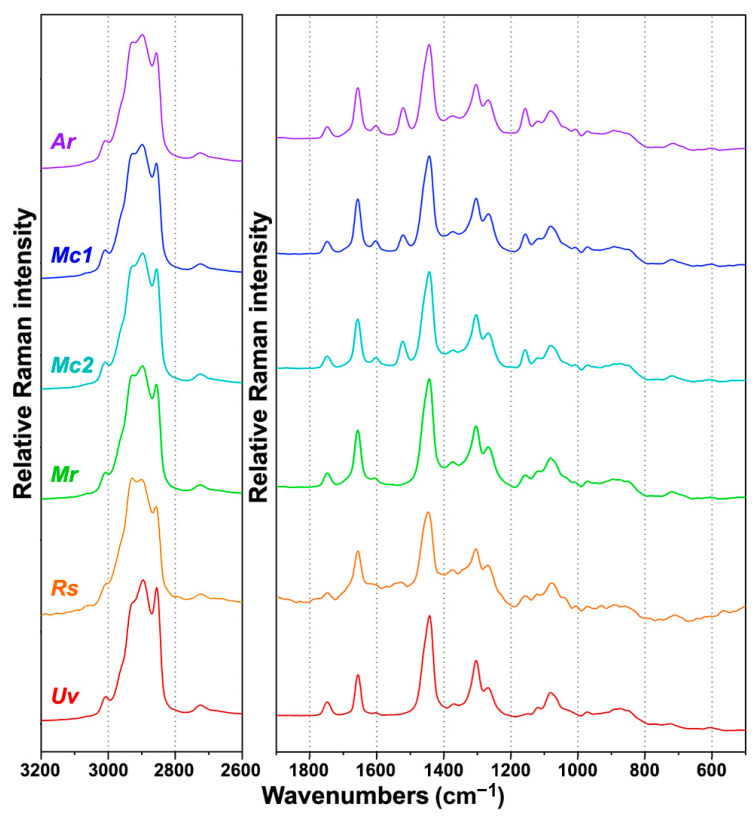
FT-Raman spectra of Mucoromycota oleaginous filamentous fungi cultivated under the standard growth condition (Ca1 and Pi1): *Amylomyces rouxii* (*Ar*), *Mucor circinelloides* VI 04473 (*Mc1*), *Mucor circinelloides* FRR 5020 (*Mc2*), *Mucor racemosus* (*Mr*), *Rhizopus stolonifer* (*Rs*), and *Umbelopsis vinacea* (*Uv*). All spectra were preprocessed and plotted with offset for better viewing.

As already mentioned, although Raman spectroscopy requires simple sample preparation and measurement, the resulting spectrum is often dominated by interference signals caused by fluorescence and sample heating. The longer wavelength excitation lasers, such as Nd:YAG laser used in this study, significantly reduce those obstructing effects. Out of 72 samples, only ten have shown interfering signals as a result of sample heating. Sample heating is a well-known problem in Raman spectroscopy, and, in this study, it was primarily caused by the absorbance of the excitation laser radiation by fungal spores. More specifically, under moderate and high phosphate concentrations, cultivations of *Rhizopus stolonifer* have resulted with slight sporulation on the walls of microbioreactor. In all cases, small presence of dark fungal spores has led to sample heating during the FT-Raman measurements, resulting in suboptimal FT-Raman spectra (Figure 3). In addition to *Rhizopus stolonifer*, the heating interference was noticeable for several *Amylomyces rouxii* samples although those samples presented no visible sporulation. In total, ten samples were measured with the reduced laser power in order to decrease the rate of heating, the heating emission spectrum, and, in particular, the sample burning. Overall, the FT-Raman measurements have resulted with high-quality spectra (Figure 4). The corresponding FTIR spectra are presented in Figure S1.

To analyze reproducibility of measurements, we used Pearson correlation coefficients (PCC) calculated for each set of the three technical replicates of FT-Raman spectra. The coefficient measures correlation between variables, where PCC value of 1 indicates high positive correlation. Therefore, small variability is indicated by small 1-PCC values. As expected, the analysis result shows that samples exhibiting heating effect, which were measured with lower laser power, have lower reproducibility (Figure S2). Nevertheless, it can be concluded that all six fungal strains were successfully measured by FT-Raman, even the highly challenging ones. In general, optimization of measurement parameters is needed when fluorescence and heating effects are present, in particular, excitation laser power and number of scans, in order to acquire quality spectra.

**Figure 5:**
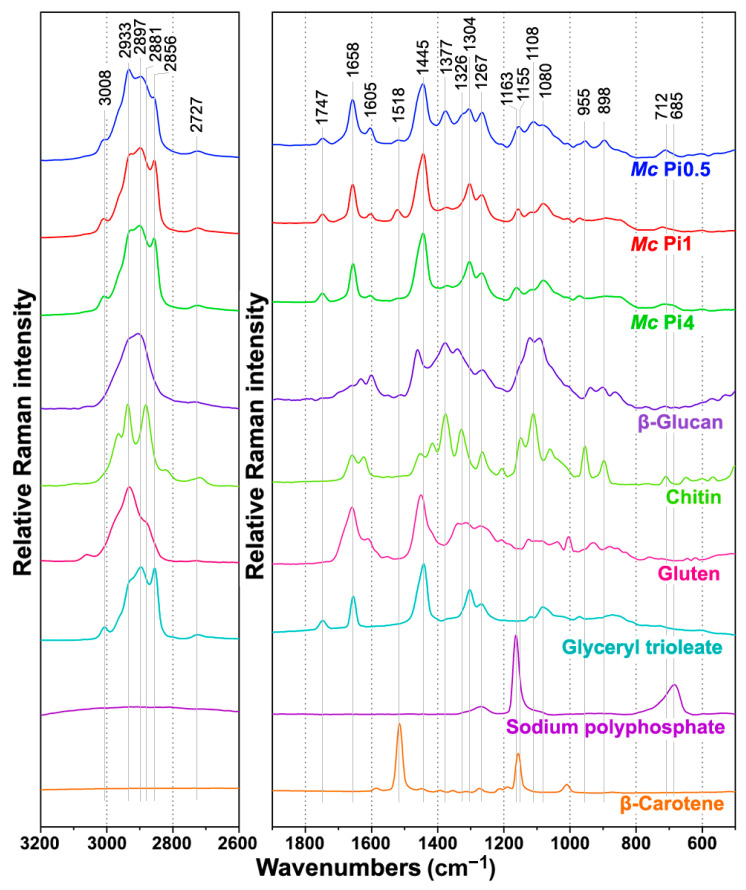
FT-Raman spectra of *Mucor circinelloides* (*Mc*) strain VI 04473 cultivated under Ca1 conditions and three different phosphate concentrations, and of six reference compounds: β-glucan, chitin, gluten, glyceryl trioleate, sodium polyphosphate, and β-carotene. All spectra were preprocessed and plotted with offset for better viewing.

FT-Raman spectra contain rich information on intracellular metabolites (Figure 5). The detailed overview of the characteristic Raman bands of main components of fungal biomass is presented in Table 1, alongside the characteristic infrared bands. In general, the most intensive Raman bands are associated with triglyceride lipids: C-H stretching vibrations (=C-H stretching at 3008 cm^−1^; C-H stretching in -CH_3_ and -CH_2_ at 2933, 2895, and 2855 cm^−1^), C=O stretching in esters (1750 cm^−1^), C=C stretching (1660 cm^−1^), CH_2_, and CH_3_ deformations (1460–1440 and 1305 cm^−1^), and C-C and C-O stretching (1080–1060 cm^−1^). In addition to the lipid-related bands, the samples show Raman bands related to cell wall carbohydrates, namely glucosamines (chitin and chitosan), glucans, and glucuronans: C-H stretching vibrations (C-H stretching in -CH_3_ and -CH_2_ at 2933, 2895, and 2885 cm^−1^), C=O stretching in esters (1755 cm^−1^, glucuronans) and amides (1680–1620 cm^−1^, Amide I, chitin), NH_2_ deformations (1620–1570 cm^−1^), CH_2_ and CH_3_ deformations (1460–1440, 1380–1320 cm^−1^), C-C, C-O, C-O-C, C-N, CH, COH stretching, deformations, and combination bands (1260–700 cm^−1^). Furthermore, minor spectral contributions were related to vibrations of proteins: C=O stretching in amides (1660 cm^−1^, Amide I), NH_2_ deformations (1620–1580 cm^−1^), phenyl ring C=C stretching (1605 cm^−1^) and deformations (1005 cm^−1^) in tyrosine and phenylalanine, CH_2_ and CH_3_ deformations (1460–1440 cm^−1^), and C-N-H deformations (1310–1250 cm^−1^, Amide III). The spectral bands associated with polyphosphates, namely P=O stretching (1165 cm^−1^) and P-O-P stretching (685 cm^−1^), were weak and barely visible in the FT-Raman spectra. This was in stark contrast to the similar bands in the FTIR spectra (at 1263 and 885 cm^−1^, respectively) that show strong absorbance.

**Figure 6:**
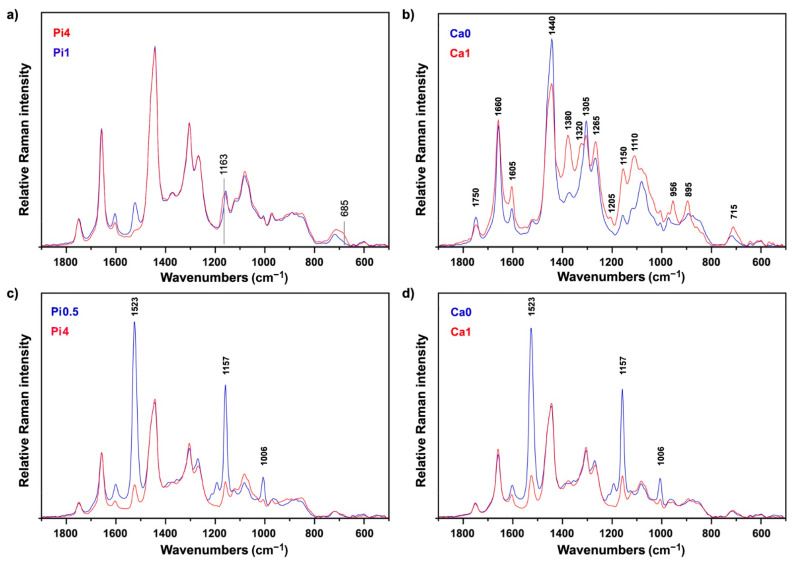
Influence of growth conditions on FT-Raman spectra of fungal biomass. Preprocessed FT-Raman spectra of: (**a**) *Mucor circinelloides* strain VI 04473 cultivated under reference calcium condition (Ca1) and two different phosphate concentrations, (**b**) *Mucor circinelloides* strain VI 04473 cultivated under low phosphates (Pi0.5) and two different calcium conditions (Ca0 and Ca1), (**c**) *Mucor circinelloides* strain FRR 5020 cultivated under absence of calcium (Ca0) and two different phosphate conditions (Pi0.5 and Pi4), and (**d**) *Mucor circinelloides* strain FRR 5020 cultivated under low phosphates (Pi0.5) and two different calcium conditions (Ca0 and Ca1).

Unlike in FTIR spectra (Figure S1), where phosphate accumulation in fungal biomass is very noticeable due to strong phosphate-related IR bands [60], phosphate accumulation in FT-Raman spectra is less noticeable. Nevertheless, these weak phosphate-related Raman bands, at 1163 and 685 cm^−1^, can be detected in the FT-Raman spectra (Figure 6a). As mentioned previously, calcium is directly involved in chitin synthesis, and thus strains cultivated in the absence of calcium show significantly lower chitin-related signals in Raman spectra when compared to their counterparts cultivated under normal calcium conditions. This is especially noticeable for samples grown under low phosphate conditions that show overexpression of chitin production as a result of acidic conditions, as exemplified by *Mucor circinelloides* strain VI 04473 (Figure 6b).

Compared to FTIR spectra of fungal biomass (Figure S1), FT-Raman spectra provide information on one additional group of chemicals: carotenoid pigments. These chemicals cannot be measured by FTIR due to their low concentration in fungal biomass. However, they can be measured with FT-Raman spectroscopy because carotenoids exhibit resonance Raman effect. In carotenoids, the conjugated nature of π-electrons from the polyene backbone causes electronic states of lower energy. Due to this, carotenoids often have absorption in the visible part of the spectrum, and they usually display strong yellow, orange and red colors (Figure 2). The resonant Raman effect causes strong enhancement of vibrational bands in carotenoids, in particular those at 1525 cm^−1^ (related to -C=C- stretching), 1155 cm^−1^ (related to -C-C- stretching and CH deformation), and 1005 cm^−1^ (related to C-CH_3_ deformations) that have strong electron–phonon coupling [75]. Out of the six studied strains, three strains show strong signals related to carotenoids: *Amylomyces rouxii* and the two *Mucor circinelloides* strains (Figure 4). These Raman bands can be used to assess influence of growth conditions on carotenoid production, and we assessed them via regression analysis based on reference carotenoid measurements (Figure 2). As visible from Figure 6c,d, high carotenoid bands are present in FT-Raman spectra of biomass of the *Mucor circinelloides* strain FRR 5020 grown in media with low phosphate concentrations and in the absence of calcium. Compared to *Mucor circinelloides* strain VI 04473, that shows overexpression of chitin, it is likely that *Mucor circinelloides* strain FRR 5020 is coping with acidic stress in the absence of calcium by overexpression of carotenoids. This is consistent with a number of studies that have shown stress related overexpression of carotenoids in filamentous fungi [76].

**Figure 7:**
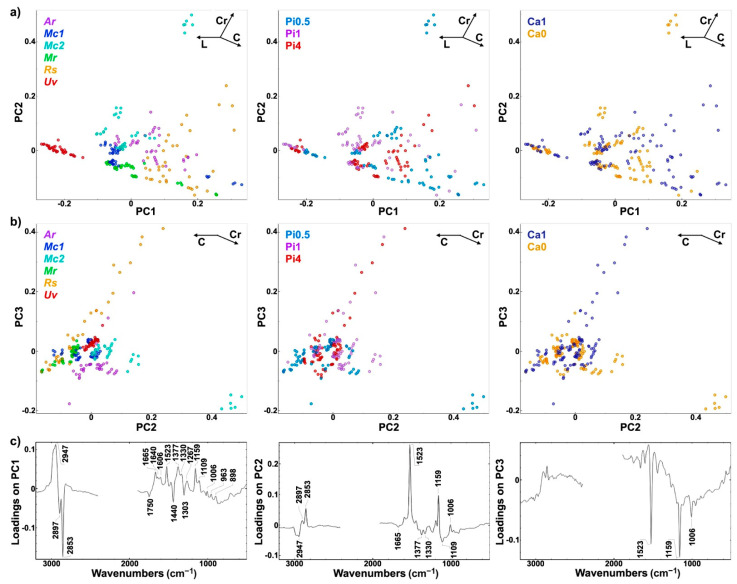
PCA of FT-Raman spectra of fungi grown at different phosphates and calcium concentrations. (**a**) Score plots of PC1 and PC2, (**b**) PC2 and PC3, and (**c**) the first three loading vectors. Score plots are labeled according to strains: *Amylomyces rouxii* (*Ar*), *Mucor circinelloides* VI 04473 (*Mc1*), *Mucor circinelloides* FRR 5020 (*Mc2*), *Mucor racemosus* (*Mr*), *Rhizopus stolonifer* (*Rs*), and *Umbelopsis vinacea* (*Uv*) (left), phosphates concentrations (middle), and calcium availability (right). Vectors are approximating the increase in relative amount of the metabolites: lipids (L), cell wall carbohydrates (C), and carotenoids (Cr). The explained variances for the first five principal components are 47.3%, 26.9%, 15.8%, 3.8%, and 1.4%.

In order to obtain general assessment of spectral variances within the whole FT-Raman spectral set, multivariate data analysis was conducted. Both FTIR and FT-Raman data are multivariate data with high collinearity. Therefore, methods based on latent variables, such as principal component analysis and partial least square regression, are often used to process such data. In PCA, graphical representations of correlations between samples, principal components, and wavenumbers allow visual detection of groups of related samples (in this case, based on sample strains and growth conditions) and consequent identification of major spectral features that are causing this differentiation. The PCA of FT-Raman data shows that the predominant spectral differences are the result of variations of bands associated with lipids, carotenoids, and cell wall carbohydrates (Figure 7). The PCA plots have high factor loadings associated with carotenoids at 1523, 1159, and 1006 cm^−1^ (positive loadings in PC1 and PC2, and negative loadings in PC3), lipids at 2897, 2853, 1750, 1440, and 1303 cm^−1^ (negative loadings in PC1), and cell wall carbohydrates (in particular chitin) at 2947, 1665, 1377, 1330, and 1109 cm^−1^ (positive loadings in PC1 and negative loadings in PC2). In particular, the signals associated with carotenoids dominate in the first three principal components. Therefore, it is evident that the FT-Raman spectral data provides complementary information to the FTIR data.

### 2.3. Quantitative Determination of Chemical Constituents of Fungal Biomass Based on Vibrational Spectra

**Figure 8:**
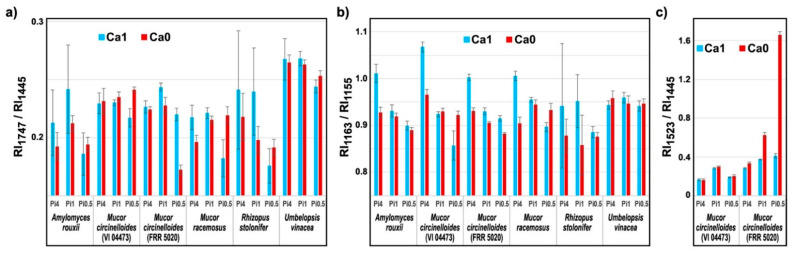
Ratio of Raman intensities at different wavenumbers related to chemical constituents of fungal biomass cultivated in six different growth conditions (phosphates concentrations and calcium availability). Ratio of Raman intensities at: (**a**) 1747 and 1445 cm^−1^ related to lipids, (**b**) 1163 and 1155 cm^−1^ related to polyphosphates, and (**c**) 1523 and 1445 cm^−1^ related to carotenoids (average values and error is based on measurements of two biological replicates and three technical replicates). Analysis was based on nonderivative FT-Raman data.

The influence of growth conditions on chemical composition of fungal biomass can be estimated by using Raman intensity ratios of Raman bands related to specific chemical constituents. Figure 8a shows that ratio of Raman intensities at 1747 cm^−1^ (related to lipids) and 1445 cm^−1^ (related to total biomass) provides a satisfactory estimate of total fungal lipids (compare to Figure 1). Ratio of Raman intensities at 1163 cm^−1^ (related to polyphosphates) and 1155 cm^−1^ (related to chitin) can be used to monitor accumulation of polyphosphates (Figure 8b), while the ratio of Raman intensities at 1523 cm^−1^ (related to carotenoids) and 1445 cm^−1^ (related to total biomass) can be used to monitor the production of carotenoids (Figure 8c; compare to Figure 2).

We demonstrated previously that PLSR of FTIR data can provide accurate assessments of intra- and extracellular fungal metabolites [58]. Therefore, quantitative estimates of total lipids, total phosphorus, and carotenoids in the fungal biomass were obtained by PLSR analyses of FT-Raman and FTIR data. The results show a high level of correlation between the vibrational data and referent measurements (Table 2 and Table 3). The RMSE values for assessment of total lipids by FT-Raman were approximately 10% for the PLSR models based on all six strains and approximately 8% for the models based on *Mucor* strains (Table 2). Similar results were obtained for FTIR-based PLSR models (Table 3), further corroborating our previous findings that FTIR spectroscopy is a practical method for quantitative analysis of total lipids in fungal biomass [57]. In general, the levels of accuracy achieved by vibrational spectroscopy PLSR models are similar to accuracy achieved by the reference method involving extraction, transesterification and chromatography.

Moreover, the RMSE values for the assessment of the total phosphorus by FT-Raman were approximately 10% for the PLSR models based on all six strains and on *Mucor* strains (Table 2). Similar results are obtained for PLSR models based on FTIR data (Table 3). Finally, the RMSE values for the assessment of total carotenoids by FT-Raman were approximately 8% for the PLSR models based on the two *Mucor circinelloides* strains (Table 2). The application of Raman spectroscopy for monitoring of carotenoids was hypothesized a decade ago, with preliminary studies on filamentous fungi *Blakeslea trispora* [12], and our results certainly confirm that quantitative analysis of total carotenoids was feasible by FT-Raman spectroscopy. The PLSR models for assessment of total carotenoids by FTIR were unstable, with large difference between prediction values of model and validation data. This is unsurprising considering that direct detection of such small content of carotenoids by FT-Raman spectroscopy was only achieved because of the resonant Raman effect, and the corresponding phenomena is not present in FTIR spectroscopy.

The number of components (PLS factors) used for building the PLSR models for both types of preprocessed FT-Raman data was low, indicating high stability and reliability of the developed models. The PLS factors clearly show contributions of relevant spectral signals, specifically signals related to lipids, polyphosphates, and carotenoids (Figures S3–S5). Moreover, it can be assumed that a large part of error of the PLS models is a result of the measurement error in the reference data and not spectral data. It is important to notice that all reference methods require large amount of biomass sample and several time-consuming processing steps involving wet chemistry. In comparison, vibrational spectroscopy methods are extremely fast and simple to implement.

### 2.4. Multiblock and Analysis of Spectral Variation by the ANOVA Model of FTIR and FT-Raman Data

**Figure 9:**
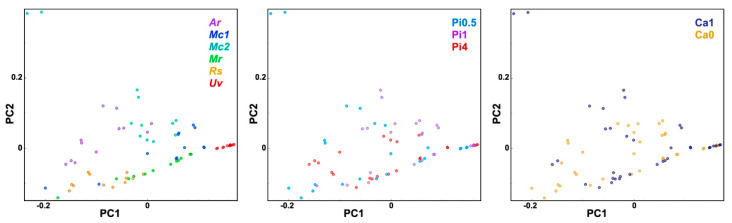
Multiblock or consensus principal component analysis (CPCA) of FTIR and FT-Raman spectroscopic data. Global score values of the CPCA are labeled according to strains: *Amylomyces rouxii* (*Ar*), *Mucor circinelloides* VI 04473 (*Mc1*), *Mucor circinelloides* FRR 5020 (*Mc2*), *Mucor racemosus* (*Mr*), *Rhizopus stolonifer* (*Rs*), and *Umbelopsis vinacea* (*Uv*) (left), phosphates concentrations (middle), and calcium availability (right). The explained variances for the first two principal components are 40.8% and 30.9%.

After averaging of technical replicates, FTIR and FT-Raman spectral sets can be analyzed as a multiblock data with a sample-to-sample correspondence between the data blocks. Consensus principal component analysis (CPCA) is a frequently used multiblock data analysis method since it allows assessment of the covariance patterns using more than one block of data [77,78]. CPCA provides global scores that describe the consensus of all data blocks involved in the CPCA. In addition, block scores and block loadings were calculated, showing individual sample and variable variation patterns for each block. Analysis of individual block scores and global scores and block loadings provides assessment of variation patterns and the molecular insights, related to sample chemistry, obtained by each of the two vibrational spectroscopic techniques (Figure 9 and Figure S6). The global scores are presented in Figure 8, and they show that the main variance is predominantly driven by variance in the FT-Raman data. The first two loadings in FTIR data block are highly correlated (Figure S6d). A similar result, with a high correlation of loadings, was presented previously on simulated data [79]. Such an effect is caused by similar variable variation patterns in one data block, while the second block shows a different effect of underlying parameters on the variable variation pattern. In our study, the reason for this is high variation in the data caused by carotenoids, which dominate variation in FT-Raman data, and are undetectable by FTIR spectroscopy. Thus, the CPCA results clearly show that FT-Raman spectra reveal additional level of chemical information about fungal biomass that is not present in FTIR data.

**Figure 10:**
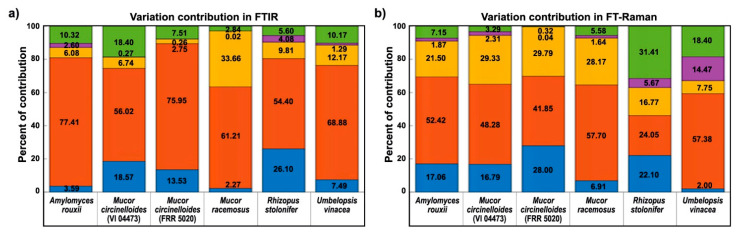
Variation contribution (%) of the design factors in FTIR and FT-Raman data sets. Spectral variation from calcium availability (blue), phosphates concentration (red), calcium–phosphates interaction (yellow), biological replicates (purple), and residuals (green) in: (**a**) FTIR and (**b**) FT-Raman spectral data (nonderivative data, averaged technical replicates).

Both spectral data sets show that phosphate concentrations have the biggest influence on the variation of biochemical profile of fungal biomass (Figure 10). This is probably related not only to the intracellular accumulation of phosphorus in the form of polyphosphates, but also to the influence of phosphate concentration on the pH of the growth media, as discussed in our previous studies [56,60,64,69]. Since the phosphate-related signals (P=O and P-O-P stretching bands) are much more prominent in the FTIR spectra than in the FT-Raman spectra (Figure 4 and Figure S2), the contribution of phosphates is higher in the FTIR dataset for strains that have significant accumulation of polyphosphates, such as *Mucor circinelloides* strain VI 04473. In general, higher contribution of calcium-phosphates interaction is present in FT-Raman data than in FTIR. Possible explanation is relatively high sensitivity of FT-Raman to the detection of changes in the chemical composition of cell wall polysaccharides and pigments, the two types of chemical constituents that are affected by both calcium and phosphates. Amongst the six cultivated strains, *Rhizopus stolonifer* was the least sensitive to different cultivation conditions (Figure 1 and [60]). Due to sporulation under higher phosphate concentrations, and the associated problems with acquiring reproducible FT-Raman spectra, this strain shows the highest residual variability in FT-Raman data. *Amylomyces rouxii* and *Mucor circinelloides* strain FRR 5020 have relatively high calcium-dependent production of carotenoid pigments (Figure 2). Since carotenoids have strong signals in FT-Raman spectra, the spectral variation due to calcium availability is higher in FT-Raman than in FTIR data. Of all the studied strains, *Umbelopsis vinacea* was able to accumulate by far the highest content of lipids. On the other hand, this strain shows no significant production of pigments and chitin/chitosan, nor accumulation of polyphosphates. Since lipid accumulation is predominantly affected by phosphates concentration, this design parameter had the highest contribution into variation in spectra of this strain in both FTIR and FT-Raman data. *Mucor racemosus* and *Mucor circinelloides* strain VI 04473 show relatively similar variation contribution profiles due to different design parameters in both spectral data sets. *Mucor circinelloides* strain VI 04473 is quite unique amongst the six cultivated strains due to significant change in polyphosphate, lipid, pigment accumulation, and cell wall chemistry, as a result of phosphate and calcium concentrations modifications. For this strain in particular, both FTIR and FT-Raman data provide valuable contribution in discerning the complex changes in biomass chemistry.

## 3. Materials and Methods

### 3.1. Fungal Strains

Six strains of Mucoromycota oleaginous filamentous fungi were used in the study: *Amylomyces rouxii* CCM F220, *Mucor circinelloides* VI 04473, *Mucor circinelloides* FRR 5020, *Mucor racemosus* UBOCC A 102007, *Rhizopus stolonifer* CCM F445, and *Umbelopsis vinacea* CCM F539. Fungi were obtained on agar slants and Petri dishes or in the lyophilized form, from the Czech Collection of Microorganisms, Brno, Czech Republic (CCM), Food Fungal Culture Collection, North Ryde, Australia (FRR), Universitè de Bretagne Occidentale Culture Collection (UBOCC; Brest, France), and the Norwegian University of Life Sciences (former Norwegian School of Veterinary Science), Ås, Norway (VI).

### 3.2. Cultivation of Fungi

Cultivation media was formulated by using the full factorial design, where three different concentrations of inorganic phosphorus substrate (high, medium, and low) and two calcium conditions (presence and absence) were used. Cultivation of the selected fungi was done in two steps: (1) growth on the standard agar medium for preparing spore inoculum and (2) growth in Duetz-MTPS in nitrogen-limited broth media with ammonium sulphate as a nitrogen source and different concentrations of the phosphorus substrate (Pi) and calcium (Ca). The cultivation in Duetz-MTPS [58] was done in two independent biological replicates for each fungus and condition, resulting in 72 samples.

For the preparation of spore inoculum, all strains except *Umbelopsis* were cultivated on malt extract agar (MEA) and *Umbelopsis* was cultivated on potato dextrose agar (PDA). MEA was prepared by dissolving 30 g of malt extract agar (Merck, Darmstadt, Germany) in 1 L of distilled water and autoclaved at 115 °C for 15 min. PDA was prepared by dissolving 39 g of potato dextrose agar (VWR, Leuven, Belgium) in 1 L of distilled water and autoclaved at 115 °C for 15 min. Agar cultivation was performed for 7 days at 25 °C for all strains. Fungal spores were harvested from agar plates with a bacteriological loop after the addition of 10 mL of sterile 0.9% NaCl solution.

The main components of the nitrogen-limited broth media [80] with modifications [56,57] were: 80 g·L^−1^ glucose, 1.5 g·L^−1^ (NH_4_)_2_SO_4_, 1.5 g·L^−1^ MgSO_4_·7H_2_O, 0.008 g·L^−1^ FeCl_3_·6H_2_O, 0.001 g·L^−1^ ZnSO_4_·7H_2_O, 0.0001 g·L^−1^ CoSO_4_·7H_2_O, 0.0001 g·L^−1^ CuSO_4_·5H_2_O, and 0.0001 g·L^−1^ MnSO_4_·5H_2_O. The concentration of calcium salt designated as Ca1, with 0.1 g·L^−1^ CaCl_2_·2H_2_O, was considered as a reference value for calcium salt, while broth media designated as Ca0 had no calcium salt present. The concentrations of phosphate salts, 7 g·L^−1^ KH_2_PO_4_ and 2 g·L^−1^ Na_2_HPO_4_, were selected as a reference value (Pi1) since they have frequently been used in the cultivation of oleaginous Mucoromycota [57,80]. The broth media contained standard concentration (designated as Pi1), higher (four times higher than the standard concentration Pi1, designated as Pi4), and lower (half of the standard concentration Pi1, designated as Pi0.5) amount of phosphate salts. Cultivation in broth media was performed in the Duetz-MTPS (Enzyscreen, Heemstede, The Netherlands), which consists of 24-square polypropylene deep well microtiter plates, low evaporation sandwich covers, and extra high cover clamps [81], which were placed into the MAXQ 4000 shaker (Thermo Fisher Scientific, Waltham, MA, USA). The autoclaved microtiter plates were filled with 7 mL of sterile broth media per well and each well was inoculated with 50 µL of spore inoculum. Cultivation was performed for 7 days at 25 °C and 400 rpm agitation (1.9 cm circular orbit).

### 3.3. Preparation of Fungal Biomass for Vibrational Spectroscopy Analyses

The growth media were separated from the fungal biomass by transferring the fermentation broth with plastic Pasteur pipettes into 15 mL Falcon tubes and the subsequent centrifugation at 13,500 rpm for 15 min at 4 °C. Fungal biomass from Falcon tubes was washed three times with cold distilled water and filtered under vacuum using a Whatman No. 1 filter paper (GE, Whatman, MA, USA). Approximately 5 mg of fresh washed biomass was transferred into 2 mL of polypropylene tube containing 250 ± 30 mg of acid washed glass beads and 0.5 mL of distilled water, and homogenized by using Percellys Evolution tissue homogenizer (Bertin Technologies, Aix-en-Provence, France) with the following set-up: 5500 rpm, 6 × 20 s cycle. Freshly homogenized biomass was measured by FTIR. The remaining washed biomass was freeze-dried for 24 h, and stored at −20 °C until FT-Raman measurements.

### 3.4. FT-Raman Spectroscopy Analysis

Raman spectra were recorded in backscattering geometry using MultiRAM FT-Raman spectrometer (Bruker Optik GmbH, Ettlingen, Germany) equipped with a neodymium-doped yttrium aluminum garnet (Nd:YAG) laser (1064 nm, 9394 cm^−1^) and germanium detector cooled with liquid nitrogen. For each measurement, 0.5–1 mg of the freeze-dried sample was deposited in an aluminum sample container and pressed with a pestle. The spectra were recorded with a total of 128 scans, using Blackman–Harris 4-term apodization, spectral resolution of 4 cm^−1^, with a digital resolution of 1.928 cm^−1^, over the range of 3785–50 cm^−1^, at a 500 mW laser power. Since some samples of *Amylomyces rouxii* and *Rhizopus stolonifer* have shown strong heating and burning effects, those samples were measured with the reduced laser power of 200 mW. Each biomass sample was analyzed in three technical replicates, resulting in 216 spectra. The OPUS software (Bruker Optik GmbH, Ettlingen, Germany) was used for data acquisition and instrument control.

### 3.5. FTIR Spectroscopy Analysis

The FTIR transmittance spectra were measured using the high throughput screening extension (HTS-XT) unit coupled to the Vertex 70 FTIR spectrometer (both Bruker Optik, Ettlingen, Germany). A total of 10 μL of homogenized fungal biomass was pipetted onto an IR transparent 384-well silica microplate and dried at room temperature for two hours. The HTS-FTIR spectra were recorded with a total of 64 scans, spectral resolution of 6 cm^−1^, and digital spacing of 1.928 cm^−1^, over the range of 4000–400 cm^−1^, and an aperture of 5 mm. Spectra were recorded as the ratio of the sample spectrum to the spectrum of the empty IR transparent microplate. Each biomass sample was analyzed in three technical replicates, resulting in 216 spectra. The OPUS software (Bruker Optik GmbH, Ettlingen, Germany) was used for data acquisition and instrument control.

### 3.6. Spectral Preprocessing and Data Analysis

All preprocessing methods and data analyses were performed using Matlab R2019a (The Mathworks Inc., Natick, MA, USA), Unscrambler 11.0 (CAMO Software, Oslo, Norway), and Orange data mining toolbox version 3.26 (University of Ljubljana, Ljubljana, Slovenia) [82,83].

#### 3.6.1. Spectral Preprocessing

Each spectral dataset (FTIR and FT-Raman) was preprocessed with two different procedures, resulting in nonderivative and derivative spectral data. For nonderivative FT-Raman data, FT-Raman spectra were smoothed by using Savitzky–Golay (SG) algorithm (polynomial 2, window size 15, derivative order 0), followed by the rubber band baseline correction, truncation of data to 3200–2400 and 1900–500 cm^−1^ regions, and normalization by extended multiplicative signal correction (EMSC), an MSC model extended by a linear, quadratic, and cubic components [84,85]. For nonderivative FTIR data, FTIR spectra were corrected and normalized by using EMSC (MSC with linear, quadratic, and cubic components). In the analysis of spectral variation due to design factors by the ANOVA model, the nonderivative data were preprocessed further, as stated below. For derivative FT-Raman data, FT-Raman spectra were converted into second derivatives by using the SG algorithm (polynomial 2, window size 15, derivative order 2), followed by the EMSC (MSC with linear, quadratic, and cubic components), and truncation of data to 1800–900 cm^−1^ region. For derivative FTIR data, FTIR spectra were converted into second derivatives by using the SG algorithm (polynomial 2, window size 15, derivative order 2), followed by the EMSC (MSC with linear and quadratic components), and truncation of data to the 1800–900 cm^−1^ region.

#### 3.6.2. Principal Component Analysis

Biochemical similarities between samples were estimated by using principal component analysis (PCA). PCA was conducted on the nonderivative spectral data. The variability test based on Pearson correlation coefficients (PCC) was used to estimate reproducibility of technical replicate measurements. The PCC test was conducted on the preprocessed non-derivative FT-Raman data. Consensus principal component analysis (CPCA) was used on multiblock spectral data, consisting of preprocessed derivative FTIR and FT-Raman data blocks. In CPCA, technical replicates were averaged after the preprocessing in order to obtain sample-to-sample correspondence between the data blocks [77,86,87].

#### 3.6.3. Quantitative Determination of Chemical Constituents of Fungal Biomass Based on Vibrational Spectra

Ratios of Raman intensities at different wavenumbers related to chemical constituents of fungal biomass (1747 cm^−1^ for lipids, 1163 cm^−1^ for phosphates, and 1523 cm^−1^ for carotenoids) were used for the initial estimation of their content. Nonderivative FT-Raman data was used for this estimation.

Partial least square regression (PLSR) was used to establish calibration models for lipids, phosphates, and carotenoids. PLSR models were established by using a data set of either GC (lipids), UV/Vis (phosphorus), or HPLC (carotenoids) reference measurements (responses) as a Y matrix, which was regressed onto an X matrix containing FT-Raman measurements (predictors). Optimal number of PLSR components (i.e., PLSR factors) of the calibration models (*AOpt*), root-mean-square error (RMSE), and coefficient of determination (R^2^) were calculated, and the optimal model was selected based on the lowest *AOpt* having insignificantly higher RMSE than the model with the minimum RMSE. PLSR analyses were conducted on both the preprocessed non-derivative and derivative FT-Raman data. PLSR models for total lipids and total phosphorus predictions were based on FT-Raman measurements of either all six fungal strains or the three *Mucor* strains, while the models for determination of carotenoids were based on the measurements of the two *Mucor circinelloides* strains. Model validation was performed by using independent biological replicates for the test set, where PLSR models were built by using one set of bioreplicate samples (bioreplicate 1) while validation was performed on the second set of bioreplicate samples (bioreplicate 2).

#### 3.6.4. Multiblock and Analysis of Spectral Variation by the ANOVA Model of FTIR and FT-Raman Data

FTIR and FT-Raman data was used to assess the influence of various experimental parameters. Spectral variation in the data introduced by the different design parameters, specifically Pi concentration, calcium availability, phosphates–calcium (Ca–Pi) interactions, and biological replicates, was calculated for each strain independently in each data set. In the analysis of variance (ANOVA) model a data matrix is represented as a sum of matrices that describe experimental design factors and the residual error. Each of these matrices consists of the means of the spectra that correspond to different levels of the design factor. The variation due to each factor can then be calculated. The ANOVA model for this study contained five design factors: calcium availability, phosphates concentration, Ca–Pi interaction, biological replicates, and unexplained residual variance. The factor “calcium availability” had two levels (Ca1 and Ca0), the factor “phosphates concentration” consisted of three levels (three different Pi concentrations), the design factor “Ca-Pi interaction” had therefore six levels, while biological replicates had two levels (bioreplicate 1 and 2). Technical replicate variations and other variations irrelevant for this study were kept as a part of residuals. The variation of each factor was normalized by the sum of the variations for the four factors of interest, meaning that they were summed up to 100%. Such an ANOVA model underlies commonly used ANOVA-PCA and ASCA analysis [88,89], which in addition to calculating variation contribution of design factors in a data allow analyzing other aspects of the data. The methods were therefore not implemented in this study. Such analysis was conducted on the preprocessed derivative FTIR and FT-Raman spectral data, where technical replicates were averaged after the preprocessing, and both FTIR and FT-Raman data were truncated to 1800–900 cm^−1^ region.

### 3.7. Reference Compounds and Reference Chemical Analyses

Details on lipid extraction and gas chromatography analysis of fatty acid profiles and total lipids and analysis of cellular phosphorus by assay-based UV/VIS spectroscopy and nuclear magnetic resonance (NMR) spectroscopy were reported previously [60].

#### 3.7.1. Reference Spectra

For chemical characterization of fungal biomass, a set of reference compounds was measured by FT-Raman and FTIR spectroscopies. Chitin, β-glucan (from *Saccharomyces cerevisiae*, predominantly β1,3-glucan linear structure with a small number of β1,6-glucan branches), gluten, glyceryl trioleate (1,2,3-tri(cis-9-octadecenoyl)glycerol), and sodium polyphosphate were purchased from Merck-Sigma-Aldrich (Darmstadt, Germany) and used without further purification.

#### 3.7.2. Carotenoid Analysis

Total carotenoid content was determined for the two *Mucor circinelloides* strains (VI 04473 and FRR 5020) by using the method based on high performance liquid chromatography equipped with a photodiode array detector (HPLC-PDA). A total of 15 ± 3 mg of freeze-dried biomass was weighed and rehydrated with 1 mL of Milli-Q water for 30 min. Excess water was removed by centrifugation at 10,000 rpm for 5 min, and 1 mL of methanol and about 0.5 mL of glass beads (0.2–0.5 mm diameter, Carl Roth GmbH, Karlsruhe, Germany) were added to the sample. The sample was vortexed for 20 min, transferred to a 15 mL tube with 2 mL of chloroform, and vortexed for 10 min. 1 mL of water was added to the sample, vortexed for 10 s, and centrifuged at 3000 rpm for 5 min. The lower (chloroform) phase was transferred to a clean tube and dried under an inert nitrogen atmosphere. The dried sample was dissolved in 1 mL of ethyl acetate: acetonitrile 1:3 and filtered through a 0.45 μm polytetrafluoroethylene (PTFE) filter into a vial. Samples were measured on Dionex Ultimate series HPLC with Vanquish diode array detector (Thermo Fischer Scientific, Waltham, MA, USA) on Kinetex C18-EVO column 150 mm × 4.6 mm × 5 µm (Phenomenex, Torrance, CA, USA) using gradient separation with mobile phase A (acetonitrile: methanol: 0.1 M tris hydrochloride pH = 8; 84:2:14) and mobile phase B (methanol: ethyl acetate; 60:40) at flowrate 1.2 mL/min and 25 °C. The following gradient program was used: 0–13 min from 100% A to 100% B, 13–19 min 100% B, 19–20 min from 100% B to 100% A, and 20–25 min 100% A. Carotenoid pigments were detected at 445 nm. Chromatographic data were evaluated using Chromeleon 7.2 software. Carotenoids were identified and evaluated using commercial standards (Sigma Aldrich, St. Louis, MI, USA) and external calibration. Only β-carotene was identified based on standards, while the remaining unidentified carotenoids were quantified via the β-carotene calibration curve.

## 4. Conclusions

The study, conducted on six strains of Mucoromycota filamentous fungi, demonstrates that quality Raman spectra of fungal biomass can be acquired by FT-Raman spectroscopy. In the case of sample heating and fluorescence, optimization of excitation laser power and number of scans is needed to reduce noise and baseline interference. FT-Raman spectra are rich in chemical information and provide data on all main chemical constituents of fungal biomass, including acylglycerol lipids, proteins, cell wall carbohydrates (glucosamines, glucans, and glucuronans), and polyphosphates. In addition, resonant Raman effect enables the detection of biomass constituents generally present in low concentrations, namely carotenoids. Effects of growth conditions (phosphorus concentration and calcium availability) on fungal biomass were clearly detectable by FT-Raman spectroscopy. Detection of fungal carotenoids, obtainable by FT-Raman and unattainable by FTIR spectroscopy, is the main difference between the two vibrational spectroscopy methods. Further, the sensitivity of the two methods in detection of other chemical constituents varies; for example, polyphosphates and proteins have strong bands in FTIR spectra and relatively weak bands in Raman spectra. PLSR models based on FT-Raman and FTIR data were established for quantitative determination of total lipids, phosphates and carotenoids. The results of PLSR analyses indicate that these vibrational spectroscopies, in combination with multivariate regression models, could be utilized as a simple, rapid, and non-destructive method for quantitative assessment of phosphorus (polyphosphates) and lipids (both FTIR and FT-Raman), and carotenoids (only FT-Raman), in intact fungal biomass.

## Figures and Tables

**Table 1:** Assignments of infrared and Raman bands: str.—stretching and def.—deformation [37,38,39,44,59,61,74,75].

Cell Component	Infrared		Raman	
	Wavenumbers (cm^−1^)	Molecular Vibration	Wavenumbers (cm^−1^)	Molecular Vibration
Carbohydrates (glucosamines, glucans, glucuronans)	3300	O-H str.	2933 and 2895	-C-H str. (CH_3_)
	3400–3100	N-H str., N-H_2_ str.	2855	-C-H str. (CH_2_, glucan)
	2879	-C-H str. (CH_3_)	1680–1620	-C=O str. (Amide I, chitin)
	1730	-C=O str. (glucuronans)	1755	-C=O str. (glucuronan)
	1680–1620	-C=O str. (Amide I, chitin)	1620–1570	NH_2_ def. (chitosan)
	1600–1550	NH_2_ def. (chitosan)	1460–1440	CH_2_ and CH_3_ def.
	1554	C-N str. & NH def. (Amide II, chitin)	1377	CH_2,_ CH, COH def.
	1375	-CH_3_ def.	1327	CH_2,_ CH, COH def.
	1305	C-N-H def. (Amide III, chitin)	1256	C-C, C-O, CH, CH_2_
	1200–1000	C-O-C str., COH def. COC def.	1200–1150	C-O-C str.
	950	-CH_3_ def.	1050–1150	C-N str. & C-C str.
			950–850	C-C str, C-O-C str. & def., COH def.
			715	O-C-O str. & CH def.
Acylglycerol lipids (triglycerides)	3010	=C-H str.	3008	=C-H str.
	2921	-C-H str. (CH_3_)	2933 and 2895	-C-H str. (CH_3_)
	2852	-C-H str. (CH_2_)	2855	-C-H str. (CH_2_)
	1743	-C=O str.	1750	C=O str.
	1463	-CH_2_ def.	1660	C=C str.
	1160	C-O-C str.	1460–1440	CH_2_ and CH_3_ def.
	723	-CH_2_ def.	1305	CH_2_ def.
			1080–1060	C-C str. C-O str.
Polyphosphates	1263	P=O str (PO_2_^-^)	1165	P=O str. (PO_2_^-^)
	885	P-O-P str.	685	P-O-P str.
Proteins	1680–1630	-C=O str. (Amide I)	1660	-C=O str. (Amide I)
	1560–1530	C-N-H def. (Amide II)	1620–1580	NH_2_ def.
	1310–1250	C-N-H def. (Amide III)	1605	C=C str. (phenyl ring)
			1460–1440	CH_2_ and CH_3_ def.
			1310–1250	C-N-H def. (Amide III)
			1005	phenyl ring def.
Carotenoids	*Not detectable at concentrations present in fungal biomass*		1525	C=C str. (polyene chain)
			1155	C-C str. & CH def.
			1005	C-CH_3_ def.

**Table 2:** PLSR coefficients of determination (R^2^) and root mean square errors (RMSE) for determination of total lipids, phosphorus, and carotenoids, with the number of components in parenthesis (*Aopt*), for the regression analyses based on nonderivative and derivative preprocessed FT-Raman data.

Analysis	Range	Nonderivative		Derivative	
		R^2^ (*Aopt*)	RMSE	R^2^ (*Aopt*)	RMSE
Total lipids (6 strains)	19.42–87.13%_dry weight_	0.83 (5)	6.60%_dry weight_	0.75 (3)	8.06%_dry weight_
Total lipids (*Mucor*)	19.42–55.57%_dry weight_	0.88 (5)	2.94%_dry weight_	0.88 (5)	2.90%_dry weight_
Total phosphorus (6 strains)	0.64–6.24%_dry weight_	0.86 (7)	0.50%_dry weight_	0.79 (5)	0.60%_dry weight_
Total phosphorus (*Mucor*)	1.40–5.20%_dry weight_	0.89 (6)	0.38%_dry weight_	0.89 (5)	0.37%_dry weight_
Total carotenoids	10.21–1669.88 µg/g_dry weight_	0.84 (1)	134.69 µg/g_dry weight_	0.84 (2)	137.34 µg/g_dry weight_

**Table 3:** PLSR coefficients of determination (R^2^) and root mean square errors (RMSE) for determination of total lipids and phosphorus, with the number of components in parenthesis (*Aopt*), for the regression analyses based on nonderivative and derivative preprocessed FTIR data.

Analysis	Range	Nonderivative		Derivative	
		R^2^ (*Aopt*)	RMSE	R^2^ (*Aopt*)	RMSE
Total lipids (6 strains)	19.42–87.13%_dry weight_	0.86 (2)	6.02%_dry weight_	0.85 (8)	6.12%_dry weight_
Total lipids (*Mucor*)	19.42–55.57%_dry weight_	0.79 (7)	3.93%_dry weight_	0.82 (5)	3.59%_dry weight_
Total phosphorus (6 strains)	0.64–6.24%_dry weight_	0.87 (9)	0.47%_dry weight_	0.84 (5)	0.53%_dry weight_
Total phosphorus (*Mucor*)	1.40–5.20%_dry weight_	0.94 (6)	0.29%_dry weight_	0.84 (4)	0.46%_dry weight_

## Data Availability

The data generated for this study are available in the Supplementary Materials.

## Supplementary Materials

The following are available online at https://www.mdpi.com/article/10.3390/ijms22136710/s1.
