# Supplementary material for: Assessment of Biotechnologically Important Filamentous Fungal Biomass by Fourier Transform Raman Spectroscopy
Source: Int J Mol Sci. 2021 Jun 23;22(13):6710. doi: 10.3390/ijms22136710 (PMC8269384; doi:10.3390/ijms22136710)
Supplement: Supplementary file 1 [file ijms-22-06710-s001.zip › Raman CaFungi_SI 02.pdf]

Supplementary Material

Assessment of biotechnologically important filamentous fungal biomass by Fourier transform Raman spectroscopy

Simona Dzurendová[1] (simona.dzurendova@nmbu.no), Volha Shapaval[1] (volha.shapaval@nmbu.no), Valeria Tafintseva[1] (valeria.tafintseva@nmbu.no), Achim Kohler[1] (achim.kohler@nmbu.no), Dana Byrtusová[1,2] (dana.byrtusova@nmbu.no), Martin Szotkowski[2] (xcszotkowski@fch.vut.cz), Ivana Márová[2] (marova@fch.vut.cz), Boris Zimmermann[1]* (boris.zimmermann@nmbu.no)

[1]Faculty of Science and Technology, Norwegian University of Life Sciences, Postbox 5003, 1432 Ås, Norway
[2]Faculty of chemistry, Brno University of Technology, Purkyňova 464/118, 61200 Brno, Czechia

Correspondence address: Faculty of Science and Technology, Norwegian University of Life Sciences, Postbox 5003, 1432 Ås, Norway

*Corresponding author:
Boris Zimmermann
Faculty of Science and Technology
Norwegian University of Life Sciences
Drøbakveien 31, 1432 Ås, Norway.
Tel: +47 6723 1576
Fax: +47 6496 5001
E-mail: boris.zimmermann@nmbu.no

Table of Contents **Page**

Figure S1. FTIR spectra of fungal biomass S-2
Figure S2. Reproducibility analysis of FT-Raman spectra S-3
Figure S3. PLSR coefficients for determination of total lipids S-4
Figure S4. PLSR coefficients for determination of total phosphorus S-5
Figure S5. PLSR coefficient for determination of total carotenoids S-6
Figure S6. CPCA score and loading plots for individual blocks S-7

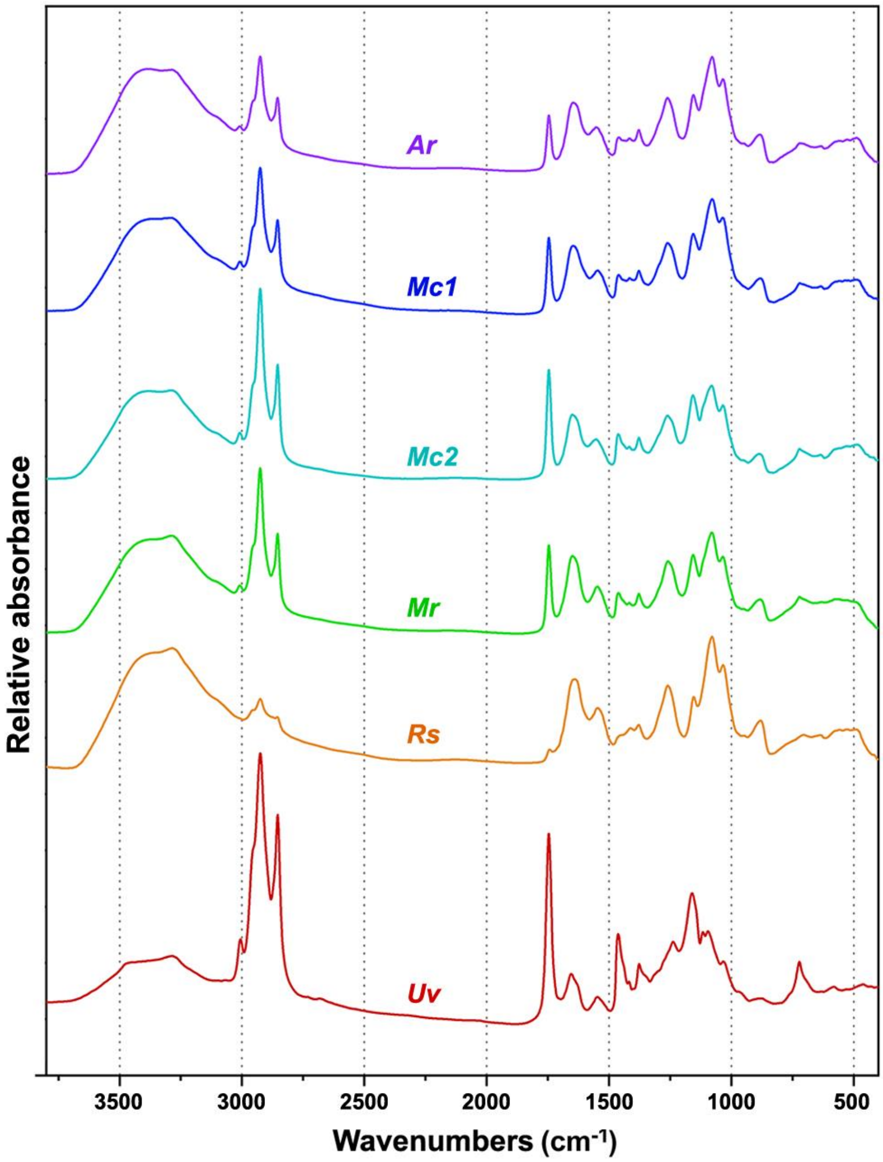

Figure S1. FTIR spectra of Mucoromycota oleaginous filamentous fungi cultivated under the standard growth condition (Ca1 and Pi1): *Amylomyces rouxii* (*Ar*), *Mucor circinelloides* VI 04473 (*Mc1*), *Mucor circinelloides* FRR 5020 (*Mc2*), *Mucor racemosus* (*Mr*), *Rhizopus stolonifer* (*Rs*), and *Umbelopsis vinacea* (*Uv*). All spectra were preprocessed and plotted with offset for better viewing.

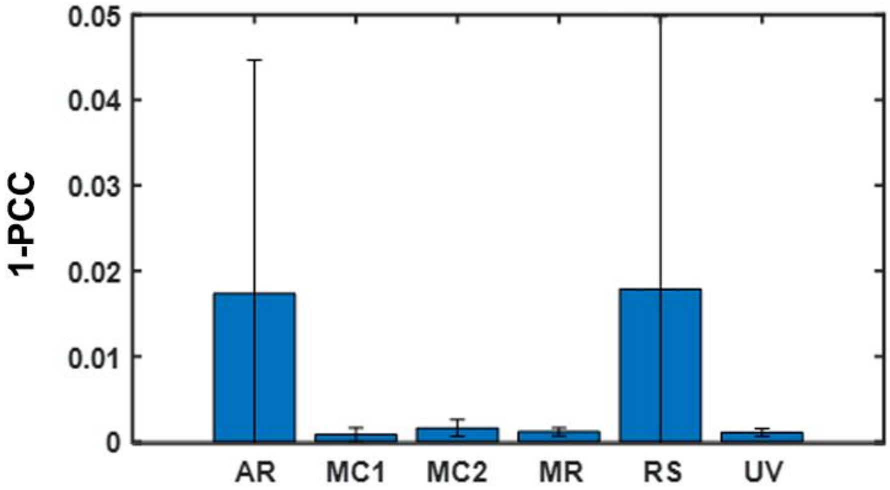

Figure S2. Reproducibility analysis of FT-Raman spectra based on PCC calculation for three technical replicates. *Amylomyces rouxii* (AR), *Mucor circinelloides* VI 04473 (MC1), *Mucor circinelloides* FRR 5020 (MC2), *Mucor racemosus* (MR), *Rhizopus stolonifer* (RS), and *Umbelopsis vinacea* (UV). Bars show 1-PCC with standard deviation. Higher bar values indicate less correlation of the technical replicates.

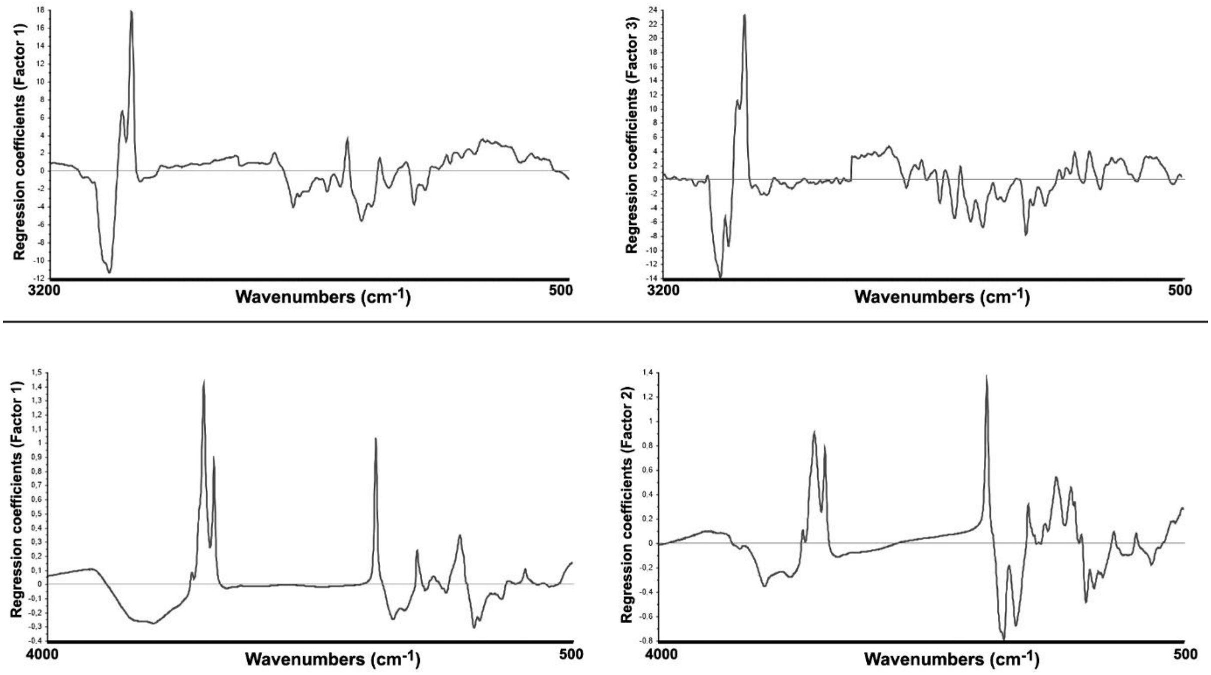

Figure S3. Plot of partial least-squares regression (PLSR) coefficients for determination of total lipids for all six strains, based on nonderivative preprocessed: FT-Raman data (up) and FTIR data (down).

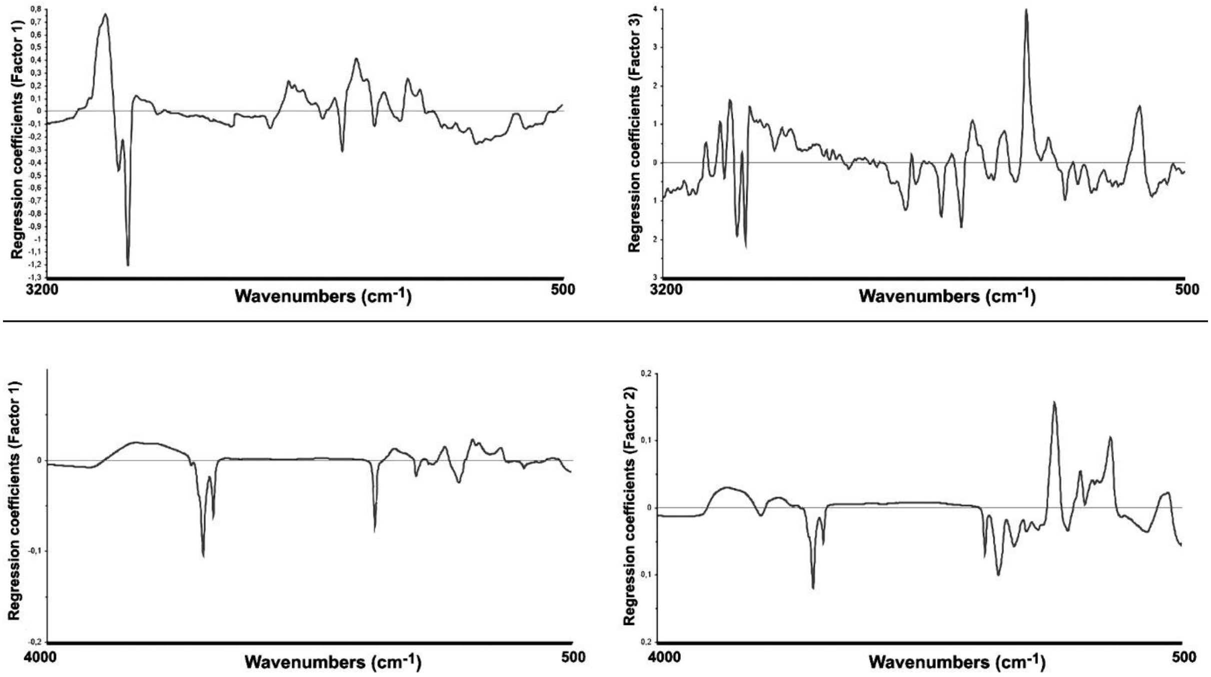

Figure S4. Plot of partial least-squares regression (PLSR) coefficients for determination of total phosphorus for all six strains, based on nonderivative preprocessed: FT-Raman data (up) and FTIR data (down).

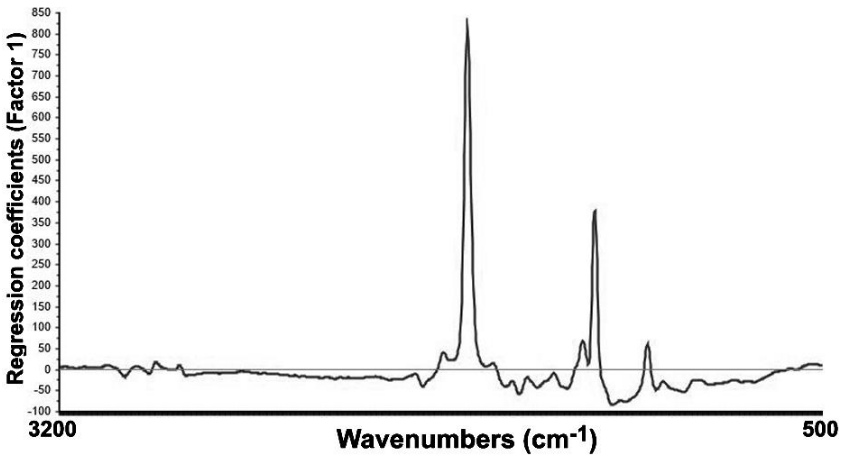

Figure S5. Plot of partial least-squares regression (PLSR) coefficient for determination of total carotenoids for *Mucor circinelloides* strains, based on nonderivative preprocessed: FT-Raman data.

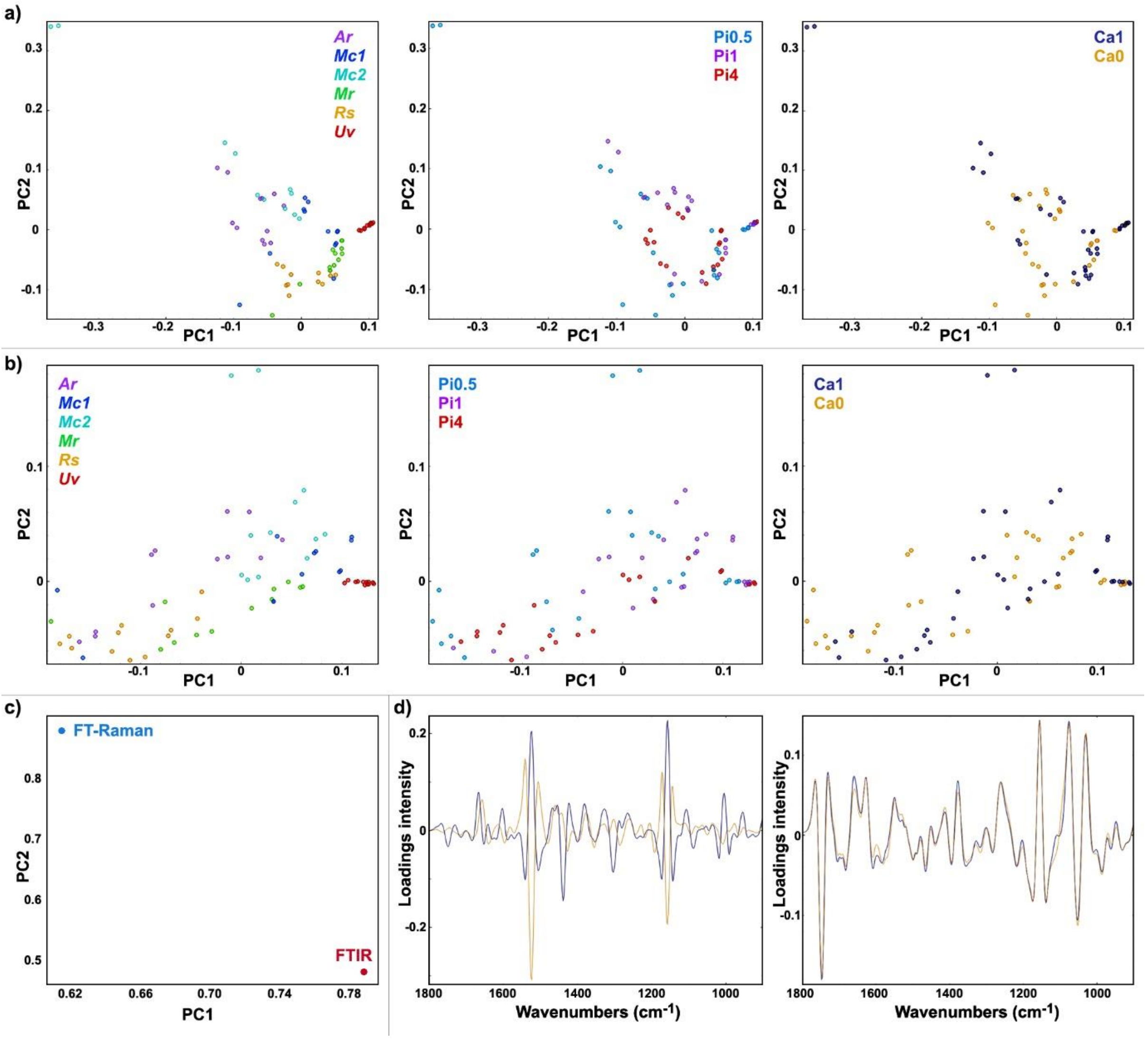

Figure S6. Multiblock consensus principal component analysis of FTIR and FT-Raman spectroscopic data. Score plots of CPCA individual blocks: (a) FT-Raman block and (b) FTIR block. Score values of individual data blocks of the CPCA are labelled according to strains: *Amylomyces rouxii* (*Ar*), *Mucor circinelloides* VI 04473 (*Mc1*), *Mucor circinelloides* FRR 5020 (*Mc2*), *Mucor racemosus* (*Mr*), *Rhizopus stolonifer* (*Rs*), and *Umbelopsis vinacea* (*Uv*) (left), phosphates concentrations (middle), and calcium availability (right). The explained variances for the first two principal components of FT-Raman block are 30.9% and 47.5%, and of FTIR block are 50.7% and 14.2%. (c) CPCA weights of individual blocks. (d) CPCA loading plots of individual blocks: FT-Raman block (bottom middle), and FTIR block (bottom right).
